# Atherosclerosis should be a rare disease in the lives of children born today

**DOI:** 10.1007/s12471-017-0976-1

**Published:** 2017-03-09

**Authors:** R. J. G. Peters

**Affiliations:** Academic Medical Center, University of Amsterdam, Amsterdam, The Netherlands

The spectacular fall in cardiovascular mortality that has occurred in most western societies in the past 40 years has contributed significantly to a steady rise in life expectancy. The Royal Dutch Actuarial Association has estimated, in its ‘Projection Table 2016’, that the life expectancy of a girl born in 2016 is 93.0 years and that of a boy born in 2016 90.1 years [1]. With a touch of optimism, these projections may in fact be an underestimation, since much of the potential for prevention of cardiovascular disease is still waiting to be used.

Consistently, the fall in cardiovascular mortality in western societies is explained as the consequence of primary prevention, improved secondary prevention after a first event, and improved acute and chronic therapy for clinical events [2, 3]. For each of these elements, further improvements may be expected, as illustrated this month in the Netherlands Heart Journal.

In this issue, current patterns of care for patients with ST-segment elevation myocardial infarction are highlighted [4]. The creation of national databases is particularly helpful in benchmarking and identifying best practices. In addition, they provide opportunities for large-scale evaluation of post-introduction changes in outcomes of interventions [5]. Together, these will assist in further improvement of clinical outcomes.

Large-scale implementation of simple and low-cost preventive therapies, such as statins, will have a major impact on the burden of disease at a population level. Statins may even provide better outcomes of acute therapies such as percutaneous coronary intervention. In this issue, an improvement of post-procedural epicardial blood flow is described in patients who are on statin therapy [6].

It is estimated that as much as 90% of the atherosclerotic disease burden may be preventable, if modifiable risk factors are adequately addressed [7]. Unfortunately, risk assessment, individual counselling and initiation of medication are all far from optimal today. An investigation of the current state of secondary prevention in Amsterdam, the Netherlands, is presented in this issue [8]. Only 4% of patients with a self-reported previous cardiovascular event had all risk factors on target. Adherence to drug therapy is far from optimal, particularly in hypertension and dyslipidaemia, in spite of a wealth of evidence on the powerful protection that it provides. Lifestyle improvements (addressing smoking, obesity, sedentary behaviour) are difficult to accomplish and even more difficult to sustain. On a societal level, education and infrastructural changes have been modest at best.

Simultaneously, powerful new therapies for dyslipidaemia are under development, such as antibodies against circulating proprotein convertase subtilisin/kexin type 9 (PCSK9). In this issue, a review is presented of current and future options for the treatment of dyslipidaemia [9]. After this manuscript was accepted, on February 2^nd^, the FOURIER trial (*n* = 27,000) was reported to show a significant risk reduction with PCSK9 inhibition (by subcutaneous evolocumab), on top of statin therapy. The main results of this trial will be presented on Friday, March 17, at *the annual meeting of the American College of Cardiology in Washington, DC.* The *Odyssey Outcomes* trial (18,600 patients), that tests alirocumab in a comparable trial design, is expected to finish by early 2018. Small interfering RNA (siRNA) molecules directed against PCSK9 synthesis may provide similar effects with less frequent dosing. However, these drugs are still in the early stages of development. Potentially, even a vaccine may be developed against PCSK9. Will a single injection early in life silence one of the most important diseases we know?

Big questions lie ahead. With life span increasing, prevention of cardiovascular disease in people above 75 years of age will be less controversial than it is today. The costs of the new classes of drugs may be prohibitive for large-scale introduction. Globally, the burden of disease may in fact increase in the coming decades, as a consequence of changing lifestyles and the process of urbanisation. Opposite trends in disease burden between affluent and developing countries would be unfortunate. Therefore, it is too early to recline and celebrate. We may have won a few battles, but the struggle continues.
